# Drug resistance characteristics and cluster analysis of M. tuberculosis in Chinese patients with multiple episodes of anti-tuberculosis treatment

**DOI:** 10.1186/s12879-015-1331-z

**Published:** 2016-01-07

**Authors:** Yi Hu, Qi Zhao, Jim Werngren, Sven Hoffner, Vinod K. Diwan, Biao Xu

**Affiliations:** 1 Department of Epidemiology, School of Public Health, Fudan University, 138 Yi Xue Yuan Rd, Shanghai, 200032 China; 2 Key Laboratory of Public Health Safety, Fudan University, Ministry of Education, Shanghai, China; 3 Microbiology and Tumor Biology Center (MTC), Karolinska Institutet, S-171 77 Stockholm, Sweden; 4 The Public Health Agency of Sweden, Solna, Sweden; 5 School of Public Health, Centre for Global Health, Karolinska Institutet, Stockholm, Sweden

**Keywords:** Tuberculosis, Multiple infection, Drug resistance, Treatment history, China

## Abstract

**Background:**

Tuberculosis (TB) patients with multiple episodes of anti-TB treatment represent an important source of TB transmission, as well as a serious threat to the control of drug resistant TB, due to the high risk of multidrug and extensively drug resistance (MDR/XDR) and elongating infectiousness of this patient group. In this study we analyzed the possible risk of development and transmission of MDR and XDR in TB patients with multiple episodes of previous treatment history.

**Methods:**

The study subjects were pulmonary TB patients who had at least two episodes of previous anti-TB treatment. A total of 166 eligible patients were identified from 10 counties/districts distributed in east, west, north, south and central China. Drug susceptibility test (DST) was performed by proportion method on LJ-media for the 1st line anti-TB drugs and a line probe assay was used to detect mutations related to resistance of the key 2nd-line drugs. Genotyping of *M. tuberculosis* (Mtb) was performed with MIRU-VNTR and Spoligotyping.

**Results:**

Resistances to 1st-line drugs was observed in 122 (73.5 %) of the 166 Mtb isolates with 97 (58.4 %) being MDR-TB. Mutations relevant to 2nd-line drug resistance was seen in 63 isolates, including 35 MDR-TB isolates (30 pre-XDR, 5 XDR-TB). The Spoligotyping revealed 83.1 % Mtb isolates belonged to the Beijing family. The MIRU-VNTR based genotyping revealed 32 (19.3 %) of patients were infected with more than one strain. The number of previous TB treatment episode was found being significantly associated with the risk of MDR-TB and XDR-TB. Among the remaining 134 patients infected with a single Mtb strain, MIRU-VNTR revealed a high homogeneity of strain especially within Beijing family despite the polymorphic variations along with geographic locations.

**Conclusions:**

The high genetic relatedness and risk of MDR-TB and subsequent pre-XDR and XDR-TB among repeatedly treated patients suggest the establishment of M/XDR Mtb in this specific patient population. It highlights the urgent needs of providing DST of both 1st- and 2nd-line drugs before and during the medication in China’s MDR-TB control program. Furthermore, the possibility of infection with multiple strains should also be considered to be associated with the drug resistance, which calls for the modification of treatment regimen.

**Electronic supplementary material:**

The online version of this article (doi:10.1186/s12879-015-1331-z) contains supplementary material, which is available to authorized users.

## Background

Multidrug-resistant tuberculosis (MDR-TB) and the even much severe form, extensively drug resistant tuberculosis (XDR-TB), has emerged as a significant global health concern. Of the world’s 12 million people living with TB, an estimated 450,000 people had MDR-TB in 2013. MDR-TB is estimated to be present in 3.7 percent of newly diagnosed patients with TB and 20 percent of previously treated patients around the world. Thus, previous treatment for TB is a strong risk factor for having MDR-TB [1]. This might be due to the treatment failure, re-infection and co-morbidity with HIV/AIDS, diabetes and other conditions [2–4]. Furthermore, the previously treated patients remain contagious for longer periods (if not fully cured). Therefore, they become one of the high risk population for transmitting resistant Mtb, especially in regions lacking effective MDR-TB control programs. Although a history of treatment is a risk factor for the development of drug resistant TB has been well recognized [5, 6], the impact of these patients on the local drug resistant-TB situation is unknown in many developing countries.

In China, of the 1,400,000 prevalent TB cases, the estimated MDR-TB rate was 5.7 % for new and 25.6 % for previously treated cases [7]. China’s TB control program provides smear microscopy-based TB diagnosis and anti-TB treatment free of charge to new patients and retreating patients who have had one full course of anti-TB treatment. Since TB patients with multiple episodes of treatment do not meet the eligibility for free TB care in China, and there are no standardization of TB medical services for patients which failed on retreatment, this population become a ‘neglected’ group [8]. However, the intricate disease profile and unclear drug resistant pattern of these patients brings about the urgent need for a timely TB diagnosis, and an individualized anti-TB treatment based on quality assured in vitro DST, which is far beyond the capacity of China’s basic TB health care units in county and district level. To accelerate proper diagnosis and treatment among patients with multiple treatment episodes, it is important to understand the occurrence of 1st- and 2nd -line drug resistance in this group of patients. Additionally, identification of major clones and its genetic characteristics in these patients will be necessary for monitoring the epidemic and transmission of drug resistant *Mycobacterium tuberculosis* (Mtb) in the communities. For this purpose, we conducted a multi-center study to investigate the drug susceptibility and genetic characterization of isolates from TB patients with multiple episodes of treatment. Meanwhile, we analyzed whether an infection with multiple strains increased the risk of drug resistance as well as possible differences in the clinical manifestation.

## Methods

### Ethical considerations

This study was approved by the Fudan School of Public Health Institutional Review Board. Written informed consent was obtained from all patients who participated in the study.

### Study design

The study subjects were smear and culture positive pulmonary TB patients who reported to have experience at least two longer than 1 month anti-TB treatment courses previously. They were identified from the patient registry system from October 2009 to February 2011 respectively from 10 counties/districts in east, west, central, north and south China. These patients are referred to designated hospitals for MDR-TB diagnosis and treatment in their respective administrative area. The eligible subjects were interviewed during their treatment course by the physician in the TB dispensaries. A structured questionnaire was used to collect the demographic and clinical information, including age, sex, HIV status, duration, regimen, tests and examinations of previous treatment, etc.

### Samples and cultures

For each patient, sputum samples were collected before the diagnosis of TB. The sputum smears were examined with smear microscopy for the presence of acid-fast bacilli. Samples were decontaminated and processed as described previously [9] using a standard N-Acetyl L-cysteine (NALC) combined with sodium hydroxide method. The processed samples were inoculated on egg-based Lowenstein Jensen (LJ) media.

### Drug susceptibility testing

Phenotypic antimicrobial susceptibilities were determined by the reference laboratory in the local designated TB hospital. The 1st-line drugs were tested using the proportional method on LJ media. The antibiotic concentrations in the medium were according to international recommendations, 0.2 mg/L for isoniazid (INH), 40 mg/L for rifampicin (RIF), 2.0 mg/L for ethambutol (EMB), and 4.0 mg/L for streptomycin (SM). Bacterial growth on drug-containing media exceeding 1 % of the number of colonies on drug-free media were considered to be resistant to the specific agent. According to the manufacturer’s protocol, the susceptibilities to the 2nd-line drugs were determined using MTBDRsl assay (Hain Lifescience GmbH, Germany).

### DNA extraction

Chromosomal DNA was extracted by centrifuging Mtb cultures and re-suspending the cell pellet in Tris-EDTA buffer. Cells were then heat-killed at 95 °C for 45 min, cooled to room temperature and centrifuged again. The supernatant containing chromosomal DNA was collected [10].

### Genetic characterization of drug resistance

Detection of mutations in the studied Mtb isolates^15^. The previously reported drug resistance related mutation was detected by DNA sequencing including the following nine genes: *rpoB* (RIF), *katG* and *inhA* (INH), *embB* (EMB), *pncA* (PZA), *gyrA* (FQs), *rrs* (KAN, CAP, and AMK), *eis* (KAN) and *tlyA* (CAP). The previously reported drug resistance-determining regions were amplified using locus-specific primers^5^. Sequence data generated by ABI 3130xl genetic analyzer were reviewed for confidence levels using an ABI sequence scanner, and chromatograms were analyzed for the presence or absence of mutations by comparison with published sequences of H37Rv using the SeqMan alignment application of the DNAStar Lasergene (version 8.0) program. All mutations were confirmed by sequencing the reverse strand.

### Spoligotyping

Spoligotyping was performed using a commercially available spoligotyping kit (Isogen Bioscience BV) in accordance with the manufacturer’s instructions. Strain family was determined by comparing spoligotyping patterns with the SpoligDB4 database. Beijing family Mtb was defined as those hybridizing the last nine spacer oligonucleotides (spacers 35 to 43) of the spoligotyping pattern.

### Typing by MIRU-VNTR

The MIRU-VNTR PCRs were performed on chromosomal DNA extracted from Mtb cultures (described above). The PCR was designed to amplify a standard set of 24 loci plus four loci (1982, 3232, 3820, and 4120) reported to be specific to the Beijing genotype strain [11]. The genomic DNA extracted from each sample using primers earlier described [12]. For each reaction, DNA from the reference strain Mtb H37Rv was used as a positive control, and sterile water was used as a negative control. PCR products were electrophoretically separated on 2 % agarose gels, using a 100-bp DNA ladder as size markers. From the gel images, the corresponding MIRU-VNTR bands were interpreted as copy numbers based on the reference table [11]. For any sample that showed multiple bands at any of the MIRU-VNTR loci, the PCR was repeated twice in order to confirm the results as well as to rule out the possibility of contamination. Multiple strains infection were noted to be present if a single sample had more than one PCR product at two or more loci, or if two or more samples from the same patient differed in copy numbers at two or more loci. Samples in which copy numbers were different at a single locus were considered to represent single strain evolution rather than multiple strains.

In combination with spoligotyping data, 24-loci MIRU-VNTR digital profiles were compared to MIRUVNTRplus (http://www.miru-vntrplus.org/MIRU/index.faces) for family and code assignment.

### Assignment to clonal complexes

Phylogenetical relationships existing between strains from different areas were investigated by drawing Minimum Spanning Trees (MST) based on 24 loci MIRU-VNTR patterns. It summarized the phylogenetic links between two MIRU profiles differing by genetic changes; the length, the color and the representation of the branches indicated the level of loci involved in changes. Thick and thin solid lines showed single and double loci changes, respectively, while dotted lines showed more than two loci changes. Different colors highlighted drug susceptibilities of strains. A clonal complex was defined by at least two strains linked by no more than two loci changes.

### Statistical analysis

ANOVA test was applied for continuous variables and Chi-square test for categorical variables. The Binary Logistic Regression Model was used to calculate the OR and 95 % CI of clustering proportion between groups with different socio-demographic and clinical features. A p value of < 0.05 was considered statistically significant.

## Results

### Socio-demographic and clinical characteristics

During the study periods, 166 previously treated TB patients whose sputum cultures were positive for Mtb were enrolled (Table 1), including 130 (78.4 %) men and 36 (21.7 %) women with an average age of 45 ± 14.7 years (range, 19 to 79 years). Seventy-eight of 166 (47.0 %) had experienced two episodes of treatment, the remained 88 (53.0 %) experienced 3–5 treatment courses. Chest radiographs showed cavitation in 69 patients (41.6 %), with highest proportion of cavity observed in patients from south areas (56.0 %). Concurrent diseases were noted in 13 (7.8 %) patients including 1 with lung cancer, 7 with respiratory diseases and 5 for diabetes mellitus according to the chart records.

**Table 1:** Socio-demographic and clinical characteristics of the 166 Chinese TB patients with multiple episodes of treatment history

	Geographic locations					
Variables	West	East	Central	North	South	Total
	*n* = 22	*n* = 29	*n* = 38	*n* = 52	*n* = 25	*n* =166
Female sex	5 (22.7)	6 (20.7)	8 (21.1)	11 (21.2)	6 (24.0)	36 (21.7)
Age (mean ± SD)	46 ± 13.8	47 ± 13.9	45 ± 17.8	46 ± 14.5	42 ± 11.8	45 ± 14.7
Episodes of previous treatment history						
=2	8 (36.4)	12 (41.4)	20 (52.6)	26 (50.0)	12 (48.0)	78 (47.0)
>2	14 (63.6)	17 (58.6)	18 (47.4)	26 (50.0)	13 (52.0)	88 (53.0)
Cavity	9 (40.9)	11 (37.9)	13 (34.2)	22 (42.3)	14 (56.0)	69 (41.6)
Concurrent disease	2 (9.1)	2 (6.9)	3 (7.9)	4 (7.7)	2 (8.0)	13 (7.8)
Drug resistant profile						
MDR only	6 (27.3)	7 (24.1)	13 (34.2)	27 (51.9)	9 (36.0)	62 (37.3)
MDR-FQR	3 (13.6)	2 (6.9)	5 (13.2)	6 (11.5)	3 (12.0)	19 (11.4)
MDR-INJR	1 (4.5)	3 (10.3)	2 (5.3)	4 (7.7)	1 (4.0)	11 (6.6)
Other 1st DR	6 (27.3)	9 (31.0)	9 (23.7)	7 (13.5)	9 (36.0)	40 (24.1)
Other 2nd DR	3 (13.6)	0 (0)	0 (0)	0 (0)	0 (0)	3 (1.8)
1st-2nd DR	2 (9.1)	7 (24.1)	8 (21.1)	6 (11.5)	2 (8.0)	25 (15.1)
XDR	0 (0)	1 (3.4)	1 (2.6)	2 (3.8)	1 (4.0)	5 (3.0)

*MDR* multidrug resistance, *XDR* extensively drug resistance, *FQR* floroquinolones resistance, *INJR* injective drug resistance

### Drug susceptibility profile and genetic mutation

By phenotypic drug susceptibility testing on the 1st-line drug and HAINs MTBDRsl assay testing on 2nd-line drug, Mtb isolates from a total of 165 (99.4 %) patients were resistant to at least one of the antimicrobial agents tested. Among 97 MDR-TB isolates, 35 isolates (36.1 %) additionally had the mutations associated with the resistance to either FQs and/or the 2nd-line injectable drugs, with five being resistant to both two groups and thus referred to XDR-TB.

Of INH resistant Mtb isolates from 116 patients, 67.2 % contained the mutation in codon 315. All the RIF resistance-related mutation were observed in the codon 531 (80.7 %), 526 (15.6 %) and 516 (3.7 %) of the *rpoB* gene. Sequencing of the *pncA* gene revealed that 39 (23.5 %) isolates had alternations including 38 non-synonymous point mutations and 1 large fragment insertions or deletions. Mutations related to FQs resistance included the mutation in codon 94 (78.0 %), codon 91 (7.3 %) and codon 90 (17.1 %) in *gyrA* gene. The 27 isolates with the mutation related to the 2nd-line injective drug resistance, contained A1401G (81.5 %), C1402T (14.8 %) and G1484T (3.7 %) of *rrs* gene.

### Strain family identification

Spoligotyping showed that Beijing family strains was the predominant group representing 83.1 % (138 of 166) of all isolates. Twenty four isolates were assigned to four other families including T family (7.8 %), CAS (3.0 %), Ural (1.8 %), H family (1.2 %) and MANU2 (0.6 %). Four strains (2.4 %) could not be classified to any earlier reported lineage thus were designated “ambiguous”.

### Distributions of patients with multiple strain infection and their clinical characteristics

Through 24 MIRU-VNTR-based genotyping plus 4 MIRU-VNTR loci specific to Beijing family, 32 of these patients were infected with multiple strains of Mtb, giving a prevalence of 19.3 % (95 % Cl: 14.0–26.0 %). Of these isolates, 12 (37.5 %) showed two alleles at one loci, 9 isolates (28.1 %) had two alleles at two loci, 6 isolates (18.8 %) had two alleles at three loci, 4 isolates (12.5 %) had two alleles at four loci, and 1 isolate (3.1 %) had two alleles at six loci.

The socio-demographic features, as well as clinical characteristics that were found in patients with a single or multiple strain were compared in Table 2. MDR-TB was found in 27 of 32 patients infected with multiple Mtb strains, which was significantly higher than those infected with a single strain (84.4 %. vs. 52.2 %, *p* = 0.001), so were the proportion of XDR-TB (9.4 %. vs. 1.5 %. *p* = 0.049). No statistically significant difference was found regarding pre-XDR although it exhibited a similar distribution between two groups with multiple and single Mtb strains (21.9 %. vs. 17.2 %, *p* = 0.534). Additionally compared to the patients with single strain infection, the patients with single strain infection had a significantly higher proportion treatment episodes more than 3 (78.1 %. vs. 43.3 %, *p* = 0.001) and cavitation (59.4 %. vs. 37.3 %, *p* = 0.023).

**Table 2:** General characteristics of patients infected with single strain or multiple strains

	Multiple strains		Single strain		P*
	*n* = 32		*n* = 134		
Social-demographic					
Age	50 ± 16.0		44 ± 14.3		0.063
Male (Sex)	23	71.9	101	75.4	0.683
BMI value	21 ± 4.2		19 ± 5.2		0.176
Clinical feature					
SS+	30	93.8	125	93.3	1
Cavity	19	59.4	50	37.3	0.023**
Treatment episode > 3	25	78.1	58	43.3	0.001**
MDR	27	84.4	70	52.2	0.001**
Pre-XDR	7	21.9	23	17.2	0.534
XDR	3	9.4	2	1.5	0.049**

*p value from the Chi-square test

**p < 0.05

### Clustering features of patient infected with single strain

**Fig. 1:**
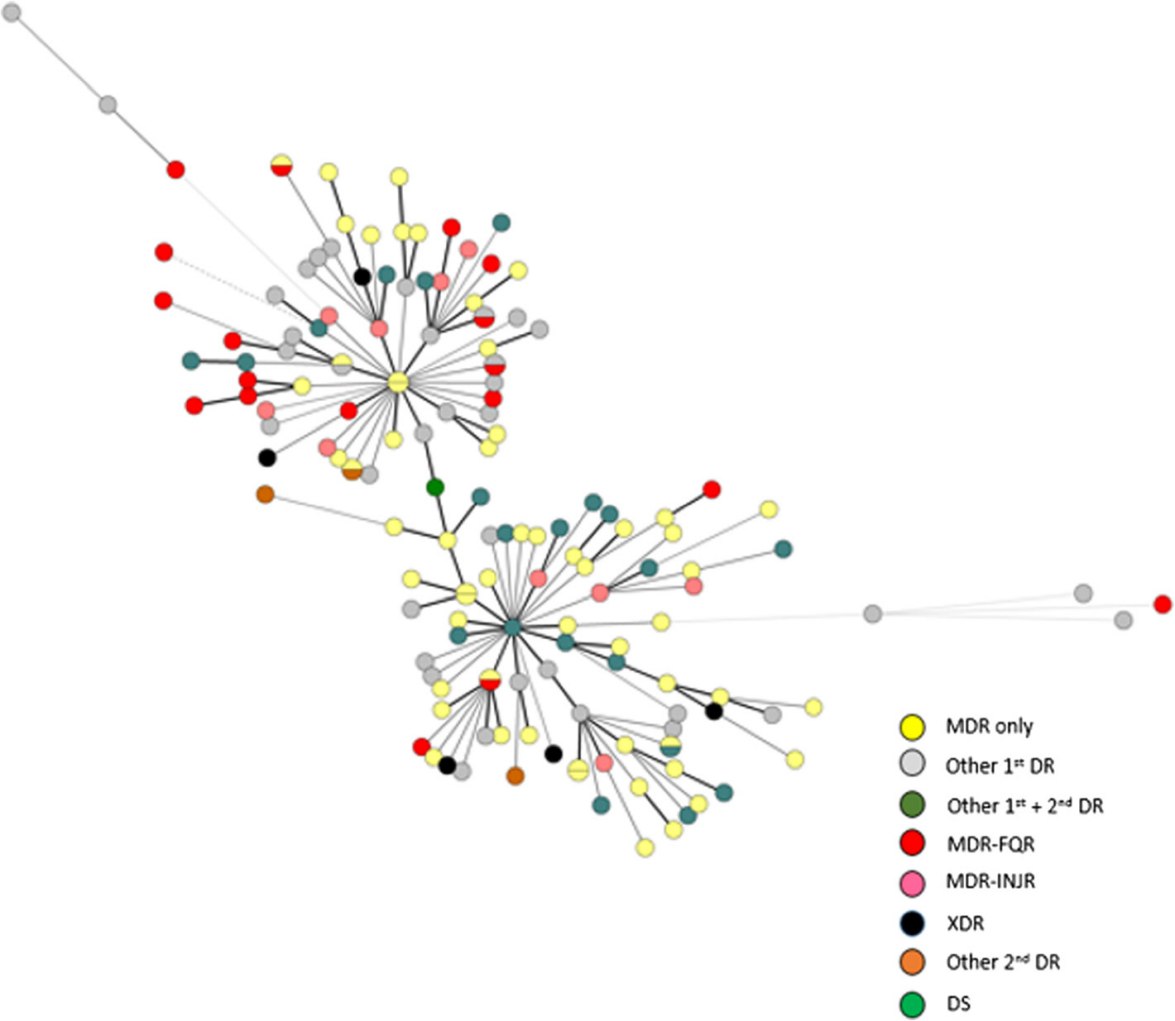
The mini spanning tree analysis of the 134 M.tb isolates from the patient with single strain infection. Each circle represents a unique genotype and a single bigger circle represents the cluster containing more than two isolates. Different color indicated the different drug resistant pattern. Note: MDR, multidrug resistance; XDR, extensively drug resistance; FQR, fluoroquinolones resistance; INJR, injective drug resistance; DS, drug sensitive; DR, drug resistance

MIRU-VNTR genotyping of Mtb isolates from the 134 patients with single strain showed that these isolates were related at ≥70 % similarity, especially for Beijing family (similarity >85 %). A slight geographic difference in MIRU-VNTR genotyping was observed, although the strain cannot be differentiated specifically to the geographic location (Fig. 1). It was also found that 101 of 134 isolates had unique MIRU types, whereas 33 isolates produced 14 clusters with identical MIRU types with three MIRU-VNTR patterns shared more than two areas.

Multivariate logistic regression analysis was applied to understand the risk factors to clustering (Table 3). Patients with three or more treatment episodes had a significantly higher clustering proportion than others (34.5 %. vs. 17.1 %; adjusted OR: 2.41; 95 % CI: 1.012–6.543). Additionally, MDR-TB patients presented a significant higher proportion of clustering than those non-MDR-TB (34.3 %. vs. 15.0 %; adjusted OR: 2.43; 95 % CI: 1.007–6.471) However, neither other socio-demographic and medical variable nor the alleles conferring resistance to 1st- and 2nd-line anti-TB drugs were significantly associated with clustering.

**Table 3:** Risk for clustering of Mtb isolates stratified by drug-resistant pattern, gene mutation and non-bacterial factors among the TB patients with single strain infection

	No. of isolates		Unadjusted		Adjusted by age, sex and areas	
	Total	Clustered (%)	OR^*^	95 % CI^*^	OR^*^	95 % CI^*^
Socio and clinical factors						
Sex						
Female	33	7 (21.2)	1		1	
Male	101	26 (25.7)	1.29	0.469–3.931	1.20	0.435–4.041
Treatment episode						
=2	76	13 (17.1)	1		1	
≥3	58	20 (34.5)	2.55	1.060–6.238**	2.41	1.012–6.543**
Sputum smear						
Negative	9	2 (22.2)	1		1	
Positive	125	31 (24.8)	1.15	0.205–11.94	1.12	0.201–12.02
Drug resistance and genetic mutation:						
Other DR	60	9 (15.0)	1		1	
MDR	70	24 (34.3)	2.45	1.008–6.363**	2.43	1.007–6.471**
preXDR/XDR	25	6 (24.0)	1.79	0.456–6.520	1.81	0.451–6.522
Drug resistant isolates with mutation in:						
WT	38	7 (18.4)	1		1	
katG	59	12 (20.3)	1.33	0.421–4.470	1.28	0.419–4.451
rpoB	55	21 (38.2)	2.74	0.943–8.622	2.64	0.873–8.953
pncA	39	8 (20.5)	1.14	0.317–4.197	1.15	0.313–4.221
gyrA	11	3 (27.3)	1.66	0.224–9.531	1.53	0.214–9.524
rrs	7	1 (14.3)	0.74	0.014–7.907	0.71	0.010–8.123
eis	4	0 (0)	-		-	

*MDR* multidrug resistance, *XDR* extensively drug resistance

^*^OR and 95 % CI were calculated in the binary logistic regression model

^**^ *p* < 0.05

### MIRU-VNTR defined clones and drug susceptibility characteristics of Mtb isolates with single strain infection

The 134 Mtb isolates from patient with a single strain were separated by Bionumerics into 11 clonal complexes (90 isolates) and singletons (44 isolates) with the default stringent definition of the groups by sharing alleles at 27 of 28 loci. The most dominant clone was comprised by 67 Mtb isolates, which all belonged to the Beijing family (Additional file 1).

The detailed information on drug resistance profiles of Mtb isolates grouped by different clones is shown in Table 4. The MDR only, pre-XDR-TB and XDR-TB was mainly observed in clone 1, while clone 2, 3, 4, 5, 6, 10 and 11 also contain one or two isolates of M/XDR-TB. In addition, we detected a large variety of drug resistance-related genes associated with the 1st and 2nd line drug resistance (such as *katG*, *rpoB*, *gyrA*, *rrs* and *eis*) in the prevalent clones.

**Table 4:** Drug susceptibilities profile and genetic mutation of the major clonal complexes in Mtb strains isolated from single infection patients

	Total.	MDR only	MDR-FQR	MDR-INJR	XDR	katG	rpoB	pncA	gyrA	rrs	eis
Clone 1	67	31 (46.3)	5 (7.5)	4 (6.0)	2 (3.0)	37 (55.2)	39 (58.2)	21 (31.3)	5 (7.5)	4 (6.0)	3 (4.5)
Clone 2	2	0 (0.0)	0 (0.0)	1 (50.0)	0 (0.0)	1 (50.0)	1 (50.0)	1 (50.0)	1 (50.0)	1 (50.0)	0 (0.0)
Clone 3	2	1 (50.0)	0 (0.0)	0 (0.0)	0 (0.0)	1 (50.0)	1 (50.0)	1 (50.0)	0 (0.0)	0 (0.0)	0 (0.0)
Clone 4	2	2 (100.0)	0 (0.0)	0 (0.0)	0 (0.0)	2 (100.0)	2 (100.0)	1 (50.0)	0 (0.0)	0 (0.0)	0 (0.0)
Clone 5	2	1 (50.0)	2 (100.0)	0 (0.0)	0 (0.0)	1 (50.0)	1 (50.0)	0 (0.0)	2 (100.0)	0 (0.0)	0 (0.0)
Clone 6	4	3 (75.0)	1 (25.0)	0 (0.0)	0 (0.0)	4 (100.0)	2 (50.0)	1 (25.0)	0 (0)	1 (25.0)	0 (0.0)
Clone 7	2	0 (0.0)	0 (0.0)	0 (0.0)	0 (0.0)	0 (0.0)	0 (0.0)	0 (0.0)	1 (50.0)	0 (0.0)	0 (0.0)
Clone 8	2	1 (50.0)	0 (0.0)	0 (0.0)	0 (0.0)	1 (50.0)	0 (0.0)	1 (50.0)	0 (0)	0 (0.0)	0 (0.0)
Clone 9	2	0 (0.0)	0 (0.0)	0 (0.0)	0 (0.0)	1 (50.0)	0 (0.0)	0 (0.0)	0 (0.0)	0 (0.0)	1 (50.0)
Clone 10	2	2 (100.0)	0 (0.0)	0 (0.0)	0 (0.0)	0 (0.0)	2 (100.0)	0 (0.0)	0 (0)	0 (0.0)	0 (0.0)
Clone 11	3	1 (33.3)	1 (33.3)	0 (0.0)	0 (0.0)	3 (100)	1 (33.3)	1 (33.3)	0 (0)	0 (0.0)	0 (0.0)
Singleton	44	11 (25.0)*	0 (0.0)*	1 (2.3)	0 (0.0)*	8 (18.2)*	7 (15.9)*	12 (27.3)	2 (4.5)*	1 (2.3)	0 (0.0)

*MDR* multidrug resistance, *XDR* extensively drug resistance, *FQR* fluoroquinolones resistance, *INJR* injective drug resistance

*p < 0.05 in comparison to the clone group

## Discussion

In our study we observed a significantly high proportion of drug resistance in the group of patients with multiple treatment courses. It was found that almost all the Mtb isolates in this study were resistant to at least one of anti-TB drugs and 58.4 % was MDR, which were much higher than that of newly treated and previously treated TB patients reported in our previous studies [7, 13, 14]. This might be due to that a high proportion of subjects have experienced more than two treatment courses. The long duration of suboptimal anti-TB treatment could impose a higher drug pressure driving the selection of drug resistant Mtb strains. The present study demonstrates a proportional association between the risk of drug resistance and number of previous treatment episodes. In addition to 1st-line drug susceptibilities, this study attempted to investigate also the 2nd- line drug susceptibilities. Interestingly, the resistance to FQs and 2nd-line injectable drugs were observed respectively in 24.5 and 16.3 % of subjects and equally distributed among the MDR-TB and other drug resistant Mtb strains. This may suggest the clinical value of the combined use of 2nd-line drug in treating the previously treated TB patients as well as the implementation of rapid and accurate DST of these anti-TB drugs.

Another important finding in this study is the high proportion of infections with multiple Mtb strains (19.3 %) among previously treated Chinese TB patients. Studies from similar high-incidence settings in South Africa showed multiple infection rates of 19 % [15], or 2.3 % using IS*6110* restriction fragment length polymorphism (RFLP) [16]. The mixed-infection patient group had the highest rate of MDR-TB, MDR plus FQs resistance and XDR-TB (Table 4). Undetected multidrug-resistant strains in a mixed infection may outcompete drug-susceptible strains during antibiotic treatment [17]. In support of this notion, mixed infection has been reported to be an important risk factor for drug resistance in a high TB-incidence region [18]. Inconsistent with an earlier reported study [18], the clinical presentations in our study appeared to be different between the patients with multiple and single strain infections. Patients infected with multiple strains exhibited more clinical symptom (cavity, smear positive) compared to those others. Since multiple strain infections could alter initial presentation or response to treatment, it should be taken into account on the clinical course for individual patients. These results indicate a lower proportion of strains sensitive to all drugs and the highest proportion of strains resistant to at least one drug in the mixed-infection group unequivocally. Thus, the mixed-infection group represents an intermediate risk group which may contribute to an increased risk of MDR. Therefore, early identification of patients with dual infections would enable prompt therapy with an optimized drug regimen increasing the cure rate.

MIRU-VNTR genotyping indicated that the degree of Mtb genotypic heterogeneity did not vary significantly between different geographical areas. This may be expected given the overwhelming dominance of the Beijing family strain in most regions in China [19, 20]. Consistent with previous reports [21–23], the results of genotyping indicate that Beijing family of strains is also overrepresented in this population. While different from the previous study [22], the member within Beijing family exhibit the close-relatedness with similarity more than 80 % and the 11 clones were identified with the majority clone comprised by 90 Mtb isolates, which all belong to Beijing genotype. This might suggest some clones within Beijing family might be responsible for the previously-treated TB. Meanwhile, the significant higher proportion of MDR-TB was observed in several clones than the others. Furthermore, all the clones within Beijing family contained 67.7 % of MDR-TB and 51.7 % of pre-XDR-TB and 33.3 % of XDR-TB, which suggest the establishment of 2nd-line drug resistant Mtb in the local setting. Therefore, Monitoring of these clones through timely genotypic analysis would provide a way to identify suspected MDR-TB and XDR-TB cases more rapidly and ensure better tracing of contacts, with the aim of containing these strains, thereby avoiding a possible XDR-TB outbreak.

The molecular typing of Mtb has greatly improved knowledge of TB epidemiology and enabled molecular guided control of the disease. The Mtb isolates within Beijing family showed a relatively high level of clustering (35.2 %), suggesting active transmission, despite no significantly difference with non-Beijing genotype. Furthermore, three Beijing MIRU-VNTR clusters were simultaneously observed in more than two areas, possibly due to its inter-provincial transmission. This tendency suggests the potential of high transmissibility for some specific members of Beijing family [17]. The active transmission of such subgroups may contribute to the ongoing epidemic of TB in rural China and highlights the need for better control measures to prevent the primary transmission. Additionally, the high proportion of clustering pattern in cases with previous treatment episode more than three times is also a warning signal that indicates that inadequate previous treatment increasing the risk of drug-resistance. Moreover, the observation of significantly high clustering proportion of MDR-TB isolates demonstrates the establishment of MDR Mtb strain transmission and its contribution to the MDR epidemics. Therefore, MDR-TB control programs in China needs additional strategies such as establishing referral system for patients at high risk of drug-resistant TB, improving ability of diagnosis for drug-resistance in TB clinics and hospitals, and providing individualized treatment for drug-resistant TB patients.

There are some limitations of this study. Firstly, the sample size might be not large enough for generalizing the conclusions. Since the TB patient with multiple treatment history is not eligible for the free treatment in China’s DOTS program, it is not possible to identify them in the TB surveillance system and therefore such a limited number of subjects were enrolled in the present study. However, despite the relatively small sample size, the multiple-centered population-based study design will ensure the representativeness of the study subjects to reflect those who were seeking TB care in the TB control program during the study period, and thus were possible to recommend to China’s MDR-TB control program. Secondly, owing to the unavailability of the culture specimen for most of subjects, we, instead, used line probe assay to estimate the proportion of 2nd-line drug resistance, which might underestimate the resistance to 2nd-line drugs in this study. However, based on the our previously reported paper [14], the gene and its genetic mutation applied in the HAINs DRsl kit proven to perform satisfactory to predicate the 2nd-line drug susceptibilities, with the sensitivity and specificity demonstrating up to 86.5 and 99.4 % respectively.

## Conclusions

This study contributes substantially to the growing body of evidence that Mtb strains from patients with multiple treatment courses exhibit a highly genetic relatedness and high risk of development MDR-TB and subsequent pre-XDR-TB and XDR-TB. These findings provide informative and fundamental evidences for better understanding the biology of tuberculosis disease and contribute to a more refined approach to control of M/XDR-TB globally, from diagnosis to treatment and prevention.

## Additional file

Additional file 1:
**MIRU-VNTR data of the Beijing family Mtb strain.** (XLSX 25 kb)

## Abbreviations

MTB: Mycobacterium tuberculosis
MDR: Multidrug-resistant
INH: Isoniazid
RIF: Rifampicin
STR: Streptomycin
EMB: Ethambutol
DST: Drug susceptibility test
